# Interictal high frequency background activity as a biomarker of epileptogenic tissue

**DOI:** 10.1093/braincomms/fcab188

**Published:** 2021-08-31

**Authors:** Truman Stovall, Brian Hunt, Simon Glynn, William C Stacey, Stephen V Gliske

**Affiliations:** 1 Department of Biomedical Engineering, University of Michigan, Ann Arbor, MI, USA; 2 Department of Neurology, University of Michigan, Ann Arbor, MI, USA; 3 Department of Neurosurgery, University of Nebraska Medical Center, Omaha, NE, USA

**Keywords:** EEG, high frequency activity

## Abstract

High frequency oscillations (HFOs) are very brief events that are a well-established biomarker of the epileptogenic zone (EZ) but are rare and comprise only a tiny fraction of the total recorded EEG. We hypothesize that the interictal high frequency ‘background’ data, which has received little attention but represents the majority of the EEG record, also may contain additional, novel information for identifying the EZ. We analysed intracranial EEG (30–500 Hz frequency range) acquired from 24 patients who underwent resective surgery. We computed 38 quantitative features based on all usable, interictal data (63–307 h per subject), excluding all detected HFOs. We assessed association between each feature and the seizure onset zone (SOZ) and resected volume (RV) using logistic regression. A pathology score per channel was also created via principle component analysis and logistic regression, using hold-out-one-patient cross-validation to avoid in-sample training. Association of the pathology score with the SOZ and RV was quantified using an asymmetry measure. Many features were associated with the SOZ: 23/38 features had odds ratios >1.3 or <0.7 and 17/38 had odds ratios different than zero with high significance (*P* < 0.001/39, logistic regression with Bonferroni Correction). The pathology score, the rate of HFOs, and their channel-wise product were each strongly associated with the SOZ [median asymmetry ≥0.44, good surgery outcome patients; median asymmetry ≥0.40, patients with other outcomes; 95% confidence interval (CI) > 0.27 in both cases]. The pathology score and the channel-wise product also had higher asymmetry with respect to the SOZ than the HFO rate alone (median difference in asymmetry ≥0.18, 95% CI >0.05). These results support that the high frequency background data contains useful information for determining the EZ, distinct and complementary to information from detected HFOs. The concordance between the high frequency activity pathology score and the rate of HFOs appears to be a better biomarker of epileptic tissue than either measure alone.

## Teaser

While high frequency oscillations are a promising biomarker of the epileptogenic zone, they comprise less than 2% of recorded data. Stovall et al., analysed the remainder of the high frequency brain activity and found that it had an equally strong association with the seizure onset zone as high frequency oscillations.

## Graphical Abstract

**Graphical Abstract:**
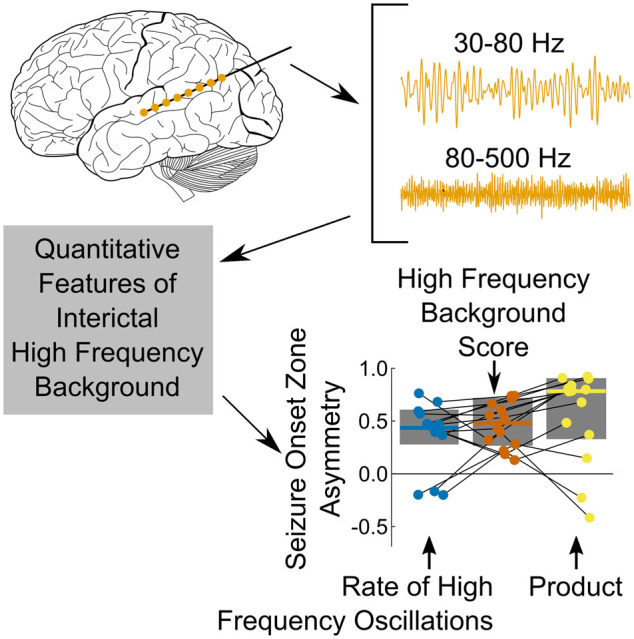

## Introduction

Epilepsy is a common neurological disorder, and about one-third of patients with epilepsy do not obtain control of their seizures with medication.^1^ One option for these patients is resective surgery. This process usually involves placement of intracranial electrodes followed by one or several weeks of inpatient monitoring to record clinical seizures. The goal is to identify whether there exists a single, focal region that can be resected to stop the patient’s seizures—the hypothesized epileptogenic zone (EZ). Clinicians consider all available information in order to determine the location of the EZ. However, the success rate of these surgeries is less than 60%.^2–4^ While many factors influence surgery outcomes, there has been great interest in identifying additional information from EEG recordings to better inform clinicians.

Interictal high frequency oscillations (HFOs) are one of the most promising biomarkers of the EZ. Multiple decades of research have firmly established the association of HFOs with the EZ, e.g.^5–13^. The desire to record HFOs has driven EEG equipment to offer higher sampling rates in modern equipment (e.g. several clinical systems now can sample ≥8 kHz, including the Cadwell Zenith, the Natus Quantum, the Nihon Kohden JE-120, and the Compumedics Neuvo). However, typical ‘high HFO rate’ channels have less than 2–10 events per minute, each lasting 20–100 ms. Thus, HFOs usually comprise from 0.04 to 1 s of data per minute (0.07–1.7%), even in the highest rate channels. Our objective in this article was to explore the remaining 99% of the high resolution data set.

Prior to this study, this non-HFO interictal high frequency data has generally been considered as background noise to be distinguished from the HFO signal and has not been directly evaluated as a biomarker of epileptic tissue or networks. This is generally true even for studies analysing complete time windows of EEG data rather than just specific, discrete events.^14–17^ Those past studies have introduced their analyses as computational shortcuts to assess the same information carried by paroxysmal, discrete events (namely, HFOs and spikes) without having to detect the events, but did not consider whether the background additionally carries other distinct information. Only one study to our knowledge considered that non-paroxysmal high frequency activity (HFA) itself may including clinically useful information,^18^ but in that case it was only in connection to phase amplitude coupling with a lower frequency signal (3–4 Hz). Here, for the first time, we assess the role of interictal high frequency (>30 Hz) activity as a potential biomarker of the EZ, separate and distinct from HFOs and agnostic to information that may be contained in lower frequencies. The objective of this article is to demonstrate that HFA is not merely noise or an epiphenomenon of detected HFOs, but that it contains novel information that is clinically relevant and deserving of future study and translation.

Our analysis of this interictal, long duration high frequency (>30 Hz) activity (which we denote HFA) involved three steps. First, we quantified the signal morphology, resulting in multiple quantitative features for each channel. Next, we conducted two independent analyses to assess the relationship between these features and best estimates of the EZ: one based on inference (quantifying the associations between each quantitative feature and clinical markings of the EZ within our dataset) and one based on prediction (leading a pseudo-prospective score of how associated each channel is with the EZ using a combination of multiple quantitative features). These two analyses thus demonstrate the association of the high frequency background activity with the EZ and yields a prototype algorithm that could be directly translated to clinical practice.

## Materials and methods

### Patient selection

Subjects were selected from patients with medically refractory epilepsy at the University of Michigan who underwent intracranial EEG monitoring in preparation for epilepsy surgery. All subjects meeting the following criteria as of April 2021 were selected: clinical data acquisition at >4 kHz sampling rate, tissue resection following intracranial monitoring, and a scored ILAE surgery outcome after at least 1 year post-resection. This resulted in a total of *N* = 24 patients, with *N* = 14 patients having Class I (ideal) surgery outcome. Subjects that were Class I when compliant with medications were considered Class I for all analyses in this article. All selected subjects had data acquired on a Natus Quantum (Natus Medical Inc.) acquisition device with a sampling rate of 4096 Hz and a 1200 Hz anti-aliasing filter. Table 1 includes further demographic and subject characteristics.

**Table 1:** Patient information

Patient	Age	Sex	ILAE Class	Resection	Pathology
UM-18	41	M	I	L Frontal Cingulate	CD
UM-19	59	F	II	R ATL	Mild gliosis
UM-20	45	F	II	R ATL	MTS within RV, PVNH outside RV
UM-21	30	M	II	R ATL	Gliosis, polymicrogyria, PVNH
UM-22	40	M	I	L ATL	Mild CD and MTS
UM-25	17	F	II	L Temporal	Gliosis
UM-28	14	F	I	R ATL	Low grade glioma
UM-30	5	M	III	L ATL	MTS
UM-31	13	M	I	L ATL (Spencer)	Gliosis within RV, NF1 outside of RV
UM-32	41	F	I	R Frontal	CD
UM-33	5	F	II	R Insula	CD, gliosis
UM-34	33	F	III	R Frontal	Gliosis
UM-35	50	F	I	L AH	Gliosis
UM-37	14	M	I	L Frontal	DNET
UM-38	28	M	II	L ATL (Spencer)	MTS, gliosis
UM-40	14	F	I	L Parietal	CD and gliosis
UM-41	32	F	I	R Frontal	CD
UM-42	17	M	II	L Insula	Not available
UM-43	28	M	II	R ATL	Gliosis
UM-46	23	F	I	L SMA	CD
UM-47	48	F	II	R ATL	Gliosis
UM-50	31	F	I	L AH	Gliosis
UM-52	27	M	I	L AH	MTS, gliosis
UM-53	55	F	I	R ATL	Gliosis
UM-54	35	F	I	L AH	Gliosis
UM-55	42	M	I	L ATL (Spencer)	HS, gliosis

AH, amygdalohippocampectomy; ATL, anterior temporal lobectomy; CD, cortical dysplasia; DNET, dysembryplastic neuroepithelial tumour; F, female; FN1, neurofibromatosis type 1; HS, hippocampal sclerosis; L, left; M, male; MTS, mesial temporal sclerosis; PVNH, periventricular nodular heterotopia; R, right; SMA, supplementary motor area.

Approval of local IRB was obtained before data collection, and all subjects/guardians consented/assented to have deidentified data be stored and analysed for future research use according to the Declaration of Helsinki. The clinically determined seizure onset zone (SOZ) was extracted from the final clinical report, written by the treating clinicians. Surgery outcome was determined by a board certified epileptologist (W.C.S.). The electrodes corresponding to resected tissue [denoted the resected volume (RV)] were determined by discussion with the neurosurgeon performing the resection and by review of the clinical data. One patient required imaging to clarify the RV due to a complicated surgery (UM-37). We used CURRY 8 (Compumedics, Charlotte, NC, USA) to coregister the CT, post-implant MRI and post-resection MRI; we then localized the electrodes, represented them as spherical volumes with radius 5 mm around the geometric centre, and computed a segmentation boundary for the RV. For this one patient, contacts were classified as within the RV if the majority of the spherical volume was on the resected side of the segmentation boundary. Further details regarding the recordings are provided in Table 2.

**Table 2:** Recording information

Patient	Total duration (h)	Analysed duration (h)	# Analysed channels (depth, ECoG)	# Channels in SOZ (depth, ECoG)	# Channels in RV (depth, ECoG)
UM-18	65.3	63.3	(32, 0)	(4, 0)	(5, 0)
UM-19	173.9	173.3	(0,106)	(0, 2)	(0, 40)
UM-20	174.1	169.3	(25, 0)	(9, 0)	(9, 0)
UM-21	185.3	178.8	(46, 0)	(13, 0)	(16, 0)
UM-22	165.3	164.8	(38, 0)	(7, 0)	(23, 0)
UM-25	211.3	206.5	(20, 0)	(5, 0)	(4, 0)
UM-28	87.5	84.1	(6, 47)	(1, 4)	(6, 12)
UM-30	151.7	123.6	(0, 100)	(0, 2)	(0, 36)
UM-31	190.7	185.7	(0, 99)	(0, 6)	(0, 54)
UM-32	187.1	158.8	(32, 0)	(3, 0)	(16, 0)
UM-33	128.0	106.3	(74, 0)	(4, 0)	(4, 0)
UM-34	144.8	129.1	(29, 0)	(11, 0)	(4, 0)
UM-35	185.0	178.2	(0, 57)	(0, 2)	(0, 11)
UM-37	232.8	212.2	(50, 0)	(7, 0)	(14, 0)
UM-38	181.9	179.8	(0, 61)	(0, 3)	(0, 30)
UM-40	203.2	199.2	(8, 55)	(0, 8)	(0, 26)
UM-41	157.3	148.3	(71, 0)	(9, 0)	(27, 0)
UM-42	73.1	73.6	(60, 0)	(8, 0)	(7, 0)
UM-43	166.6	144.6	(86,0)	(9,0)	(30,0)
UM-46	141.5	128.7	(30, 0)	(9, 0)	(12, 0)
UM-47	306.9	306.5	(70, 0)	(3, 0)	(30, 0)
UM-50	179.8	174.4	(22, 71)	(0, 3)	(0, 16)
UM-52	137.2	144.2	(0, 61)	(0, 3)	(0, 10)
UM-53	188.6	187.8	(68, 0)	(3, 0)	(28, 0)
UM-54	185.3	174.7	(0, 61)	(0, 9)	(0, 14)
UM-55	236.5	234.4	(62, 0)	(7, 0)	(27, 0)

**TOTAL**	4,440.9	4,229.7	(827, 718)	(115, 39)	(262, 249)

Recordings were obtained from all times of day. We provide the number of channels separately for depth electrodes and those on the cortical surface, i.e. electrocorticography (ECoG) electrodes.

### Computation of features

#### Preprocessing

The preprocessing steps included restricting to interictal data, excluding obvious data with poor quality, and re-referencing. Only interictal data (at least 30 min before or after the start of a clinical seizure) were included in the analysis and results. Seizure times were obtained from the clinical reports. Data with ambiguities in seizure times, and channels with obvious extremely poor quality or known to be extraparenchymal were excluded from the analysis. Data were also excluded during and near times coinciding with EEG technical care and testing procedures. All interictal data not excluded by the above steps were analysed. We used a common average reference as in previous work,^9^ and again separate references were used for depth and grid/strip channels.

#### Frequency ranges

While there is no clear standard regarding what constitutes ‘high’ frequency, we decided to use two frequency bands. We selected one frequency band to correspond with the typical frequency band used for detecting HFOs, 80–500 Hz. We note the exact frequency band for HFOs varies across the literature and that there are inconsistent findings regarding the utility of separating ripples (80–250 Hz) from fast ripples (250–500 Hz). We thus selected 80–500 Hz for our upper band, the same range used in our previous HFO work, e.g. Gliske et al.^9^^,^^19^ and Ren et al.^20^ We then selected a second frequency band to span the range between the frequencies typically viewed by clinicians and this range, resulting in a selection of 30–80 Hz. This selected band has been denoted the low-gamma band and included within the broad range of HFO frequencies, e.g. Jrad et al.^21^ Ongoing research supports the involvement of this frequency in epileptic brain activity, e.g. Zweiphenning et al.^22^ These bands were previously shown to have distinct mechanisms in computational modelling work: postsynaptic potentials due to normal gamma in the 30–80 Hz range, and strongly driven postsynaptic potentials and/or pyramidal cell action potentials in the 80–500 Hz range.^23–26^ We also ran the analysis on a subset of patients (the first 10 ILAE Class I subjects) using three bands, 30–80 Hz, 80–250 Hz and 250–500 Hz (data included in online repository, see section ‘Data availability’), and found qualitatively similar results and no particular advantage. When filtering data, we used a 10th order, bidirectional elliptical passband filter, with a 0.5 dB passband ripple and 65 dB stopband ripple, all parameters except the pass band range being the same as in previous work.^9^

#### Feature quantification

We next sought to quantify the morphology of the high frequency EEG waveform. We note two approaches are common in the literature for developing mathematical features to describe electrophysiological signals. One is to develop a small set of strongly motivated features specifically designed to assess known aspects of the signal. A second approach is to develop a large set of features that describe many aspects of the signal and then to use dimensionality reduction and machine learning to identify the most useful combinations of features. Both approaches have their merit. Since the goal of this article is to identify aspects of the HFA signal associated with epilepsy—the very information needed to take the first approach—the second approach is optimal for this article.

Features were computed per channel in epochs of 5-min duration. HFOs and artefacts were detected using a previously validated algorithm,^9^ and all data corresponding to HFO detections and artefacts were removed from each 5-min epoch. Thus, some epochs use less than 5-min of data to compute the features, due to redaction of HFOs and artefacts. A total of 38 features were then computed, 19 per frequency band. Each feature was computed by applying a transformation to the data, followed by computing a statistical measure, and lastly a scaling function. The specific choices for transformation and statistical measures were selected to be very general. We used statistical measures related to the first four statistical moments of a random variable, which assess the position, spread, asymmetry and bluntness of the distributions, and we used four transformations that are common in time-series analysis: absolute value, transformations related to the first and second derivatives, and the Teager-Kaiser Energy operator. The full set of features are listed in Table 3. Features are computed per each channel and each epoch, and we enumerate the set of all channel/epoch combinations with the index *i*. We represent the vector of values of the filtered waveform for the *i*-th channel/epoch combination a*s*
 

(1) $$x^{\left(i\right)}={\left\{x_{j}^{\left(i\right)}\right\}}_{j=1}^{n},$$

where *n* is the total number of samples and each $x_{j}^{\left(i\right)}$ represents a specific sample value. With the exception of one feature, all features (see Table 3) were computed by normalizing the data via subtracting the mean and dividing by the standard deviation. The normalized vector was thus

(2) $$y^{\left(i\right)}={\left\{\frac{x_{j}^{\left(i\right)}-\mu \left(x^{\left(i\right)}\right)}{\sigma \left(x^{\left(i\right)}\right)}\right\}}_{j=1}^{n}\;,$$

where μ(·) and σ(·) represent computing the mean and standard deviation. The following transformation include factors of the sampling rate in kHz, which we denote $f_{S}$. We used four transformation operators in computing features, the rectification operator *R*, the Line-Length operator *L*, the curvature operator *C*, and the Teager-Kaiser Energy operator *T*:

(3) $$R\left(y^{\left(i\right)}\right)={\left\{\left|y_{j}^{\left(i\right)}\right|\right\}}_{j=1}^{n},$$

 

(4) $$L\left(y^{\left(i\right)}\right)=R\left(D\left(y^{\left(i\right)}\right)\right)={\left\{\left|\left|y_{j+1}^{\left(i\right)}-y_{j}^{\left(i\right)}\right|f_{S}\right|\right\}}_{j=1}^{n-1},$$

 

(5) $$C\left(y^{\left(i\right)}\right)=R\left(D\left(D\left(y^{\left(i\right)}\right)\right)\right)={\left\{\left|\left|y_{j+2}^{\left(i\right)}+y_{j}^{\left(i\right)}-2y_{j+1}^{\left(i\right)}\right|{\left(f_{S}\right)}^{2}\right|\right\}}_{j=1}^{n-2},$$

 

(6) $$T\left(y^{\left(i\right)}\right)={\left\{\left({\left(y_{j}^{\left(i\right)}\right)}^{2}-y_{j+1}^{\left(i\right)}y_{j-1}^{\left(i\right)}\right){\left(f_{S}\right)}^{2}\right\}}_{j=2}^{n-1}$$

**Table 3:** Feature definitions

	$10{\;\text{log}\;}_{10}\left(\mu \left(\cdot \right)\right)$	$10{\;\text{log}\;}_{10}\left(\sigma \left(\cdot \right)\right)$
$x^{\left(i\right)}$		$f_{5}^{\left(i\right)}=10\;{\;\text{log}\;}_{10}\left(\sigma \left(x^{\left(i\right)}\right)\right)$
$R\left(y^{\left(i\right)}\right)$	$f_{1}^{\left(i\right)}=10\;{\;\text{log}\;}_{10}\left(\mu \left(R\left(y^{\left(i\right)}\right)\right)\right)$	$f_{6}^{\left(i\right)}=10\;{\;\text{log}\;}_{10}\left(\sigma \left(D\left(y^{\left(i\right)}\right)\right)\right)$
$L\left(y^{\left(i\right)}\right)$	$f_{2}^{\left(i\right)}=10\;{\;\text{log}\;}_{10}\left(\mu \left(L\left(y^{\left(i\right)}\right)\right)\right)$	$f_{7}^{\left(i\right)}=10\;{\;\text{log}\;}_{10}\left(\sigma \left(L\left(y^{\left(i\right)}\right)\right)\right)$
$C\left(y^{\left(i\right)}\right)$	$f_{3}^{\left(i\right)}=10\;{\;\text{log}\;}_{10}\left(\mu \left(C\left(y^{\left(i\right)}\right)\right)\right)$	$f_{8}^{\left(i\right)}=10\;{\;\text{log}\;}_{10}\left(\sigma \left(C\left(y^{\left(i\right)}\right)\right)\right)$
$T\left(y^{\left(i\right)}\right)$	$f_{4}^{\left(i\right)}=10\;{\;\text{log}\;}_{10}\left(\mu \left(T\left(y^{\left(i\right)}\right)\right)\right)$	$f_{9}^{\left(i\right)}=10\;{\;\text{log}\;}_{10}\left(\sigma \left(T\left(y^{\left(i\right)}\right)\right)\right)$

	${\;\text{tan}\;}^{-1}\left(\text{skew}\left(\cdot \right)\right)$	$10{\;\text{log}\;}_{10}\left(\text{kurt}\left(\cdot \right)\right)$

$y^{\left(i\right)}$	$f_{10}^{\left(i\right)}={\;\text{tan}\;}^{-1}\left(\text{skew}\left(y^{\left(i\right)}\right)\right)$	$f_{15}^{\left(i\right)}=10\;{\;\text{log}\;}_{10}\left(\text{kurt}\left(y^{\left(i\right)}\right)\right)$
$R\left(y^{\left(i\right)}\right)$	$f_{11}^{\left(i\right)}={\;\text{tan}\;}^{-1}\left(\text{skew}\left(R\left(y^{\left(i\right)}\right)\right)\right)$	$f_{16}^{\left(i\right)}=10\;{\;\text{log}\;}_{10}\left(\text{kurt}\left(D\left(y^{\left(i\right)}\right)\right)\right)$
$L\left(y^{\left(i\right)}\right)$	$f_{12}^{\left(i\right)}={\;\text{tan}\;}^{-1}\left(\text{skew}\left(L\left(y^{\left(i\right)}\right)\right)\right)$	$f_{17}^{\left(i\right)}=10\;{\;\text{log}\;}_{10}\left(\text{kurt}\left(L\left(y^{\left(i\right)}\right)\right)\right)$
$C\left(y^{\left(i\right)}\right)$	$f_{13}^{\left(i\right)}={\;\text{tan}\;}^{-1}\left(\text{skew}\left(C\left(y^{\left(i\right)}\right)\right)\right)$	$f_{18}^{\left(i\right)}=10\;{\;\text{log}\;}_{10}\left(\text{kurt}\left(C\left(y^{\left(i\right)}\right)\right)\right)$
$T\left(y^{\left(i\right)}\right)$	$f_{14}^{\left(i\right)}={\;\text{tan}\;}^{-1}\left(\text{skew}\left(T\left(y^{\left(i\right)}\right)\right)\right)$	$f_{19}^{\left(i\right)}={\;\text{log}\;}^{-1}\left(\text{kurt}\left(T\left(y^{\left(i\right)}\right)\right)\right)$

Features are computed by starting with either the normalized ($y^{\left(i\right)}$) or non-normalized ($x^{\left(i\right)}$) data per for each epoch/channel combination *i*; c.f. Equations 1 and 2. Next, a transformation is applied (none, rectification *R*, line-length *L*, curvature *C*, or Teager-Kaiser Energy *T*; c.f. Equations 3–6), followed by a statistical measure (mean, variance, skewness or kurtosis). Lastly, a scaling transformation (arctangent or conversion to decibels) is applied. Rows in the table represent specific transforms to the data, and columns represent specific combinations of statistical measure and scaling transformation.

**Figure 1:**
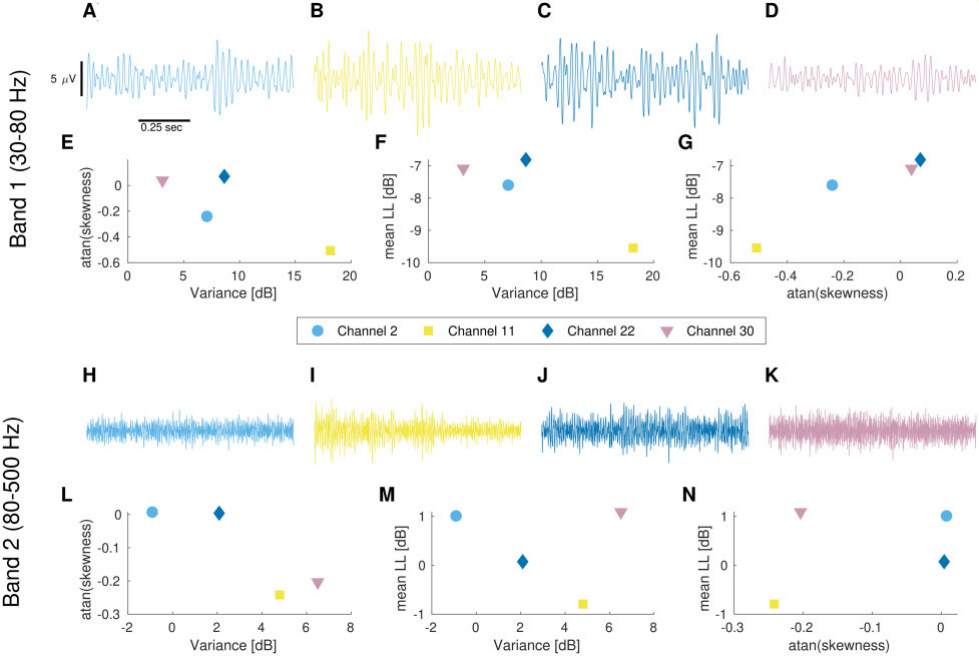
**Example raw data and associated quantitative features.** Example intracranial EEG data recorded concurrently from four channels in patient UM-18 (**A**–**D**, **H**–**K**). Additionally, scatter plots of three of the quantitative features for the associated 5-min epoch for each of three pairwise comparisons (**E**–**G**, **L**–**N**). Note, all data in the upper panels (**A**–**G**) correspond to Band 1 (30–80 Hz), while all data in the lower panels (**H**–**N**) correspond to band 2 (80–500 Hz). The scalebars shown in (**A**) are applicable for all EEG traces (**A**–**D**, **H**–**K**). The channels for each trace are (**A and H**) channel 2, (**B and I**) channel 11, (**C and J**) channel 22, (**D and K**) channel 30. Note, the widely varying feature values are not readily apparent by visual inspection of the filtered data. Furthermore, the full time-integrated features used in the analysis (see section ‘Reduction to time-integrated features’) include comparisons of the relative feature values across channels for a 5-min epoch of high temporal resolution data (more data than can be viewed on a single screen), as well as comparisons across all 5-min epochs. Thus, the information assessed in the high frequency background in this manuscript is beyond that which is extractable by human review.

The factors of sampling rate in kHz result in the features having units which are powers of voltage per msec rather than voltage per 244.14 µs inter-sample duration (244.14 µs is the inverse of 4096 Hz). While not directly pertinent to this analysis, this normalization removes the inter-sample duration from the units, which is a necessary step for the features to generalize across different sampling rates. In order to reduce the heavy tails of the feature distributions, units for all features not involving skewness were converted to units of decibels (10 times the logarithm base 10). As skewness involves negative values, converting to decibels would lead to complex numbers. We thus instead used the arctangent, which accomplishes the same goal of log-scaling the heavy tails but is applicable for both negative and positive data. The means in Table 3 were computed using Kahan sums for numerical stability, with the Boost C++ library being used to compute the sums, standard deviations, kurtoses, and skewnesses. Example filtered data and some corresponding quantitative features are shown in Fig. 1. Note that many changes in quantitative features were not readily apparent by visual review.

#### Reduction to time-integrated features

**Figure 2:**
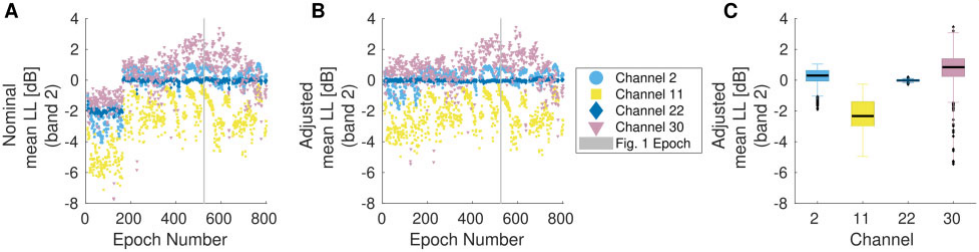
**Correction for temporal variability.** (**A**) Mean of the line length transformation applied to 80–500 Hz filtered data as originally computed for each 5-min epoch on four example channels from subject UM-18. An abrupt change in the value of this feature occurs around epoch 180. (**B**) The same feature, but adjusted by subtracting the median value per epoch (computed over all channels). The abrupt shift near epoch 180 is greatly reduced. Note, the epoch from which data in Fig. 1 was drawn is indicated by a grey vertical line in panels **A and B**. (**C**) Box plot of the feature data in panel (**B**). The upper and lower box edges are drawn at the first to third quartiles, with whiskers extending out to the last data point within one interquartile distance from the nearest box edge. Outliers are shown as small black diamonds. Note, the time-integrated value for this feature (the value corresponding to that which is used for the remaining analysis) is the third quartile (upper edges of the boxes).

Thus far, we have computed quantitative features per each 5-min epoch of each channel. Using these individual epochs, we then calculated a single value of each feature for each channel per subject. This is analogous to taking the individual HFO detections over time and converting to a single HFO rate per channel. In order to reduce the effect of temporal variability, our first step was to subtract off the median value within each 5-min epoch; see Fig. 2A and B. Then, for each channel, we considered the distribution of each adjusted feature over all epochs and selected the location of the 75th percentile. We denote these values as the time-integrated features since they incorporate information across the full recording duration; see Fig. 2C. These time-integrated features are then used for both the internal association and prediction analyses.

### Internal association analysis

The internal association (inference) analysis sought to determine whether features of the high frequency background are associated with clinical markers of epileptogenic tissue (the SOZ and RV). A standard approach is to use logistic regression, which we accomplished by using the MATLAB *fitglm* function. Since the features are correlated, it is necessary to compute a logistic regression model for each feature to assess its individual utility. We also computed separate models for each outcome variable, the SOZ and RV, as well as for the temporal lobe. Temporal lobe asymmetries were included to check for a possible anatomical confounding factor. The anatomical locations of electrodes (Destrieux Atlas) for 21/24 subjects had been computed for other projects using Freesurfer. This information was then used to identify which contacts were in temporal lobes. Weights were introduced so that each patient contributed equally to the model, rather than each channel contributing equally. This approach better accounts for possible interpatient variability in the features as well as the variety in the number of channels per patient. For example, among these 24 patients, the number of intracranial channels ranged from 20 to 106 (see Table 2). In order to compare results across features that are in different units, we divide each feature by its scaled median absolute deviance (sMAD) before computing the model. The sMAD was computed using the MATLAB *mad* function with flag = 1, with the result divided by the inverse cumulative normal distribution function evaluated at 0.75. The sMAD was selected instead of standard deviation, as the distributions are not normal and channels with feature values in the heavy tails are exactly those we are trying to identify. The result is an odds ratio and confidence interval (CI) for each feature, describing the relative change in the odds of the channel being in the SOZ (or RV) for each increase of the feature by one sMAD. For example, the difference between a channel with a feature at the first quartile and a channel with a feature at the third quartile would be one sMAD. We also computed a similar model for the HFO rate, for comparison. The odds ratio and CI can be interpreted as an effect size. The logistic regression also provides a *P*-value for testing the hypothesis that the effects are not random (i.e. that the odds ratio is not one).

### Predictive analysis

While the internal association analysis can demonstrate utility of the features, to actually translate this utility into practice requires a predictive analytic approach. To avoid in-sample training, we used hold-out-one-patient cross-validation. We repeated the following procedure 24 times (as *N* = 24 patients), using each patient’s data alone one time for testing, where each time the training data were taken from all patients except the one being tested. The data were whitened by subtracting the mean value and dividing by the standard deviation, with mean and standard deviation computed using just the training data. We then reduced the dimensionality using principle component analysis (PCA, Matlab *pca* function), selecting enough features to capture 95% of the variance. Again, the PCA subspace was computed using just the training data and then applied to the testing data. Lastly, logistic regression was used to compute a predictive model of the likelihood an individual channel is within the SOZ based on the training data, using the PCA components as the independent (input) variables and inclusion in the SOZ as the dependent (output) variable. SOZ was selected as the dependent variable rather than RV as it is generally more specific to the epileptogenic tissue and networks. Applying the logistic regression model to the testing data in this PCA space then results in a score per channel, which we denote the pathological high frequency activity score (pHFA). This score ranges from a value of 0 (least likely to be pathological) to 1 (most likely to be pathological), though the relative score across channels is likely more informative than the absolute magnitude.

To assess the predictive value of this score, we computed the asymmetry with respect to SOZ and RV, as was done for HFO rates in previous analyses.^9^^,^^19^^,^^27^ We also computed the asymmetry with respect to the temporal lobe to assess the magnitude of this potential confounding factor. The clinically-determined SOZ and RV in patients with ILAE Class I surgical outcomes are the best available estimates of the EZ. The asymmetry was computed by first averaging the pHFA score over all channels within (denoted *x*_in_) and without (denoted *x*_out_) the SOZ (alternately the RV or temporal lobe), and then computing the asymmetry as *A* = (*x*_in_-*x*_out_)/(*x*_in_ + *x*_out_). We also computed the asymmetry of the HFO rate using the qHFO detector.^9^ Noting that HFOs and the pHFA score tended to agree most on channels within the SOZ and RV and agree less on channels without, we also created a hybrid HFO/HFA score by multiplying the HFO rate and the pHFA score for each channel. Using multiplication to combine the two scores has the advantage of not being sensitive to the different scale of the units of the two measures and of dampening the signal in all channels except those where both signals are high, analogous to a logical ‘and’. The asymmetry for this product was also computed for the SOZ, RV and temporal lobe. The main results are based on the effects sizes, computed as the median asymmetries for each case and the median change in asymmetries, along with the 95% CI for each of those quantities.

### Data availability

Raw data were recorded at the University of Michigan. Full source data cannot be posted due to its massive size: over 250,000 channel hours of data at 4096 samples per second were analysed (3.7 trillion samples). Derived data and Matlab scripts supporting the findings of this study are available at https://doi.org/10.7302/hnvs-f543 Accessed 27 August 2021. Specifically, we include the data and metadata starting just one step after the source data: the 38 quantitative features for every channel for every 5-min epoch of data (38 × 3,026,493 total feature values). All figures and results of the paper can be reproduced from the posted information. Note, we used Matlab revision 2020b.

## Results

### Internal association analysis

**Figure 3:**
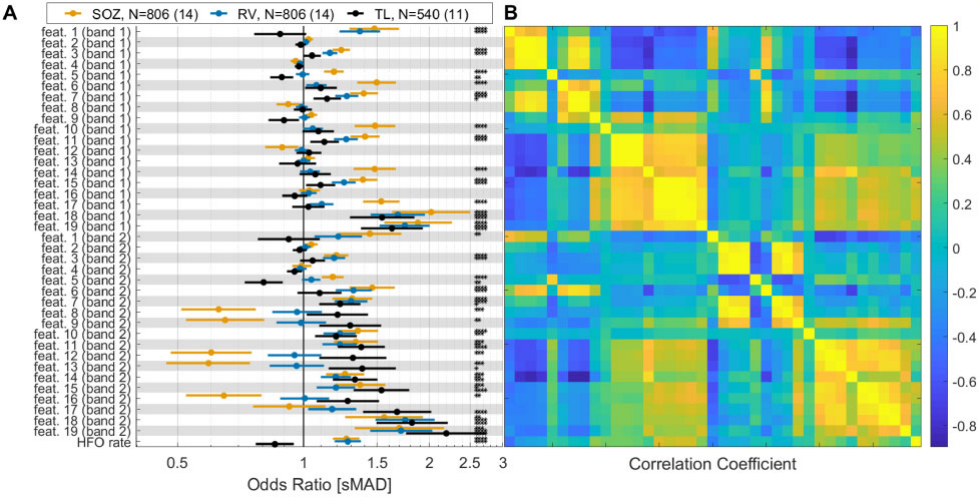
**Internal association results.** (**A**) The odds ratio for each feature (logistic regression) is presented for the association with seizure onset zone (SOZ), resected volume (RV) and temporal lobe (TL). HFO rate is also included for comparison. Band 1 is 30–80 Hz and band 2 is 80–500 Hz. See Table 3 for definition of features: feat. 1 corresponds to $f_{1}^{}$, feat. 2 corresponds to $f_{2}^{}$, etc. *P < 0.05/39; **P < 0.01/39; ***P < 0.001/39; ****P < 0.0001/39 (logistic regression with Bonferroni correction for multiple comparisons). The number of channels (*N*) is given for each comparison type, with the number of patients given in parenthesis. The displayed odds ratio of about 1.5 for the SOZ model for feature 1 means that an increase of this feature of one scaled median absolute deviance (sMAD) implies the channel would be 1.5 times more likely to be in the SOZ. (**B**) Correlation coefficients between features are presented in the same order as in (**A**), with HFO rate included for reference. Specific test-statistics and *P*-values are included in the data repository (see section ‘Data availability’).

The internal association analysis assessed the relationship between each feature and the SOZ, RV and temporal lobe within the available data. Results are shown in Fig. 3A. The effect sizes relative to the SOZ were large for many features: 23/38 features had odds ratios >1.3 or <0.7. Additionally, 28/38 features had odds ratios statistically different than zero (*P* < 0.05/38, logistic regression with Bonferroni correction for multiple comparisons), and 17/38 had *P* < 0.001/38. The relationship with RV was generally slightly weaker, as expected: 6/38 had odds rations >1.3, 13/38 features had odds ratios statistically different than zero at the level of *P* < 0.05/38, and 12/38 with *P* < 0.001/38. The lower association with RV than SOZ is not surprising, as the RV typically includes all or most of the SOZ, plus several other channels that were resected for anatomical considerations that may not be in the EZ. A number of features were also associated with the temporal lobe, though the pattern of which features were informative appear qualitatively distinct from the pattern for SOZ and RV (Fig. 3A). Note, many features have an odds ratio similar or better than that for the HFO rate (last row in Fig. 3A). The correlation between features is shown in Fig. 3B for reference. HFO rate is also correlated with some features, especially those involving kurtosis or skew in the higher frequency band, even though the HFO data was not used. Overall, the results show that the high frequency background activity carries significant information about the location of the EZ, with the odds ratios having comparable or superior values to the HFO rate.

### Predictive analysis

**Figure 4:**
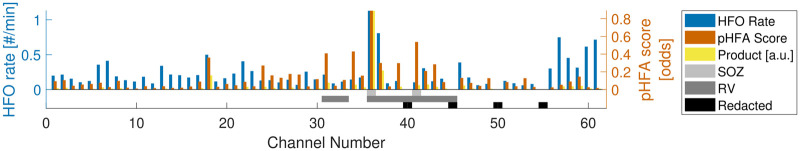
**Example HFO rate and pHFA-score per channel.** Data are shown for subject UM-35, with the procedure to compute the pHFA score (principle component analysis and logistic regression) being trained on the other 23 subjects. The qHFO rate is also shown for reference. Also indicated are channels redacted during preprocessing (channels which were extraparenchymal or had extremely low signal quality, see section ‘Preprocessing’). The product of the HFO rate and pHFA score is shown in arbitrary units (a.u.) for visualization. Although the HFO rates were not extremely specific in this case, the pHFA score is clearly higher in the two SOZ channels (channels 36 and 41), and the channel-wise product of HFO rate and pHFA score is highly specific to one SOZ channel (channel 36).

The predictive analysis included computing a pseudo-prospective score per channel of how pathological the HFA was, i.e. the time-integrated pHFA score. Note, the score is based on the distribution of background features relative to other channels within the same patient (see section ‘Reduction to time-integrated features’)—aspects not readily apparent in visual review. An example patient (UM-35) is shown in Fig. 4. This patient had a parietotemporal grid (channels 1–35), a basotemporal grid (channels 36–55) and an anterior temporal strip (channels 56–61). The channel with the highest HFO rate (channel 36, the most anteromesial contact of the basotemporal grid) was one of the two SOZ channels (the other SOZ channel being channel 41, immediately posterior to channel 36). However, the HFO rate in this subject was difficult to assess: the rate in channel 36 was not much higher than several other electrodes which were not resected (e.g. 57 and 61). The pHFA score was high in both of the SOZ channels. However, it was also relatively high in other channels, specifically some in the anteromedial corner of the basotemporal strip, near the SOZ channels, and some in the middle of the anterior temporal strip. The product of HFO rate and pHFA score, in contrast, was very specific: highlighting just one channel of the SOZ. Thus, taking the product of pHFA score and HFO rate resulted in dampening the background HFO rate and pHFA score and focusing the prediction on the channel in which both were high.

**Figure 5:**
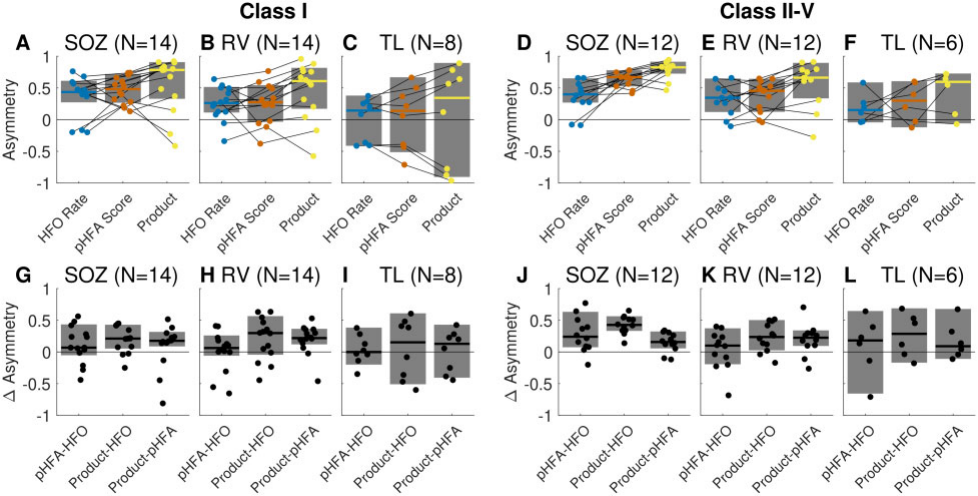
**Asymmetry values.** (**A**–**F**) Asymmetry values per patient are shown along with the median value (colour bars) and 95% confidence level (grey rectangles). Asymmetries were computed with respect to the seizure onset zone (SOZ), resected volume (RV) and temporal lobe (TL) for three biomarkers: the HFO rate, the pathological high frequency activity (pHFA) score and the channel-wise product of the two (denoted as ‘Product’ in the figure). Black lines connect the asymmetry value for each given patient. Results are shown separately for patients with ideal surgery outcome (ILEA Class I, **A**–**C**) and less than ideal surgery outcome (ILEA Class II–V, **D**–**F**). (**G**–**L**) The difference in asymmetry values between pairs of biomarkers shown along with the median value (black bars) and 95% confidence level (grey rectangles). Results are shown separately for patients with ideal surgery outcome (ILEA Class I, **G**–**I**) and less than ideal surgery outcome (ILEA Class II–V, **J**–**L**). Asymmetry scores are a measure of effect size, and results for which the 95% confidence bands do not include zero are statistically significant. Presenting results using effect sizes is in contrast to using statistical tests and *P*-values. The number of subjects per subject varies across categories and is indicated in the figure next to the panel letter.

To assess the association of this score with the SOZ, RV and the temporal lobe across all patients, we computed asymmetry scores per patient; see Fig. 5. The median asymmetry was greater than zero in all cases (central bars in Fig. 5A–F), with the 95% CI being quite positive for all comparisons relative to the SOZ. The comparison with RV was not quite as strong, with the lower 95% confidence bound being less positive (and in one case slightly negative). In contrast, 95% CI for comparison with the temporal lobe (Fig. 5C and F) always included zero, indicating no statistically significant association. For each patient, we additionally computed the difference in asymmetry scores (Fig. 5G–L) to assess relative utility of one biomarker versus another. We observed that in both cohorts, the product of pHFA score and HFO rate was better than either marker alone. We also observed the pHFA score was not inferior to the HFO rate, with some patients having the pHFA score asymmetry being much higher than the HFO rate asymmetry. Results thus support that the pHFA score, like HFO rate, has prospective utility for identifying EZ.

## Discussion

This article presents a method to analyse the high frequency, interictal EEG background activity distinct from HFOs and low frequency activity, and demonstrates that this signal (the pHFA score) has strong association with the SOZ and RV using both internal association and predictive analytic approaches. While this score was acquired on the same high-sampling rate intracranial EEG data that are used for HFO studies, the pHFA score assesses the data very differently. For example, pHFA does not necessarily require any specific detector—it is merely a measurement on the interictal background. We nonetheless found two distinct advantages to including the HFO detector in our analysis: (i) by redacting HFO detections, we were able to clarify that the background itself has intrinsic utility; and (ii) we found that concurrence between HFO detections and the background pHFA score was a better biomarker than either alone. Similar to our qHFO algorithm,^9^ the pHFA score is easily translatable, requiring minimal human input on uncurated clinical data. Since our results demonstrated that the pHFA score has utility distinct from and complementary to HFO rate, the results thus suggest that pHFA is a unique biomarker of epileptic tissue worthy of more research and development.

Since one of the goals of the article was to show the relevance of HFA activity itself (distinct from that of HFOs), we specifically redacted all detected HFOs before computing HFA features. However, in some situations, it might be preferable to compute HFA features without having to identify and redact HFOs. We reran the analysis on a subset of patients (the first 10 ILEA Class I subjects) two additional times: once with redacting artefacts but not HFOs and once with not redacting either artefacts or HFOs (data included in the online repository). We observed qualitatively that the results were markedly worse when including artefacts, and subtly improved when including HFOs. Thus, we strongly recommend using artefact detectors when working with HFA.

To our knowledge, our work is the first to present and support HFA as a unique biomarker, distinct from HFOs. Our work is different than using background data to set thresholds for HFO detection (e.g. Staba et al.^28^), to determine optimal times for HFO detection (e.g. Zelmann et al.^29^), or as surrogate for HFO detection.^14–17^ While we specifically excluded detected HFOs, it is possible that some undetected HFOs still contaminated our signal. However, even if we assume that the number of undetected HFOs is as large as the number of detected HFOs, they would span less than 2% of the analysed data and would be relatively low amplitude. When comparing to the effect of adding in all HFOs to the analysis (data included in the online repository), we conclude that it is highly unlikely that the utility of the pHFA score is only due to it including some non-detected HFOs.

The objective of this article was to demonstrate that HFA contains novel information that is clinically relevant and deserving of future study. Our results show that HFA is highly associated with epileptic tissue and that it provides additional information beyond what is provided by HFO analysis. In contrast to HFOs, these data are not dependent upon specific detectors, or other nuances of detecting discrete events. Thus, we conclude that HFA is a promising new biomarker of epileptic tissue, complementary to HFOs. Future work will be necessary to assess how this information, both HFO and HFA, influences clinical decisions about the SOZ and surgical planning.

We note that one limitation of our study was the inability to fully optimize all decisions made in the analysis given the relatively small number of subjects. For example, a number of parameters were set *a priori* and we then analysed and reported the results of the choices. These included the epoch duration (5 min), the number and range of the frequency bands (30–80 Hz and 80–500 Hz), and the percent variance for PCA. This lack of optimization is not necessarily a weakness but a strength: despite not optimizing these values, we still observed strong association of HFA features with SOZ and RV. We also reran the analysis on a subset of patients (the first 10 Class I patients) using shorter and longer epoch durations (2 and 10 min), with a different number of frequency bands (using 30–80 Hz, 80–250 Hz and 250–500 Hz), and with percent variance thresholds of 90% and 97% (data included in online repository discussed in section ‘Data availability’). We found the results generally quite robust to variation of these parameter values, with insignificant changes in 9/10 patients and significant changes in just one patient for two of the different parameters. Further optimization of these parameters, including the frequency bands, could potentially increase the utility of HFA analysis.

The limited number of subjects prevents us from addressing possible confounding factors, such as anatomical location or specific pathology. Instead, we have reported the available information in Tables 1 and 2 to be clear regarding potential impact. We additionally compared the biomarkers within and without of the temporal lobe, the most likely anatomical confounding factor. Some quantitative features of the high frequency background were found to be characteristic of the temporal lobe (Fig. 3). However, these features were distinct from those that were most informative for identifying the SOZ, as the trained pHFA score was not overly specific to the temporal lobe (Fig. 5C and F).

Some of the previous work using analysis of high-frequency background EEG as a surrogate for HFO detections has focused on features related to the general concept of ‘spikiness’. Features include the kurtosis,^14^ skewness^16^ or both.^17^ We found these features were still associated with SOZ and RV even when redacting all HFOs. Thus, results in these previous publications likely benefitted from information contained in both the HFOs and in the general, long-duration high frequency background activity, which we call HFA. Expanding on these previous publications, in our results we found many new features associated with SOZ and RV in addition to skewness and kurtosis. Although correlations exist between evaluated features, these additional features add information and are not all redundant. We note that the derivatives of the signal used in the transforms place an increased focus on the higher frequency components of the signal. Furthermore, our results suggest that the usefulness of HFA cannot just be reduced to one or two properties: in all 24 cross-validation folds, the PCA procedure identified at least 9 linear combinations of features as necessary to capture 95% of the variance. Future work in larger data sets can further isolate whether a specific subset of features is sufficient or whether the conglomerate feature approach is preferable.

We designed the features and analysis to identify abnormal channels. We also found it was necessary to remove channels that were extraparenchymal and or that had extremely poor data quality (see section ‘Preprocessing’) before applying the analysis. Note that these poor quality channels are those that are ignored by clinicians. The difference between good and bad quality data is large and confounds the ability to identify abnormality due to epileptic tissue. The feasibility of using HFA in clinical settings could be enhanced by automating the selection of good quality channels, though we note that clinicians often identify poor quality channels and times with unreliable data as part of the standard of care. The amount of data curation used for our methods is quite small. We also note that HFOs were studied for decades before automated methods to handle variable data quality were developed.^9^

One of the essential aspects of our analysis was subtracting the mean of the features within each time epoch (see section ‘Reduction to time-integrated features’). Thus, the analysed features and pHFA score per channel are implicitly measures relative to other channels in the same patient. Without this normalization across channels, temporal variability causes the pHFA score to have much lower association with the SOZ and RV (data not shown). This may be another factor explaining why pHFA score is not readily visible: one must compare across all channels at once. Instead, the computed pHFA score by design mitigates the influence of temporal variability. While beyond the scope of this manuscript, we anticipate that pHFA score will have less temporal variability than HFO rate but will still require several hours to days of recordings, not just a few minutes. Recall that the variability in HFO rate is not only due to apparent randomness, but also that subsets of seizure networks seem to turn on and off over the course of days.^27^ Similarly, HFA may also be sensitive to these changing seizure networks, and thus short recordings will miss such information.

In many patients, determining the EZ is challenging. In these cases, clinicians often gather all available data to look for concordance. Our analysis found that concordance between HFO rate and pHFA score, specifically their product, was an even better biomarker than HFO rate alone. However, when comparing with the broader set of heterogeneous information (seizure onset and spread, imaging, semiology, etc.), the skill and experience of the clinical team is essential. Thus, like HFOs, the pHFA rate is not expected to replace current clinical practice of determining SOZ, but our results show that it can add adjunctive, relevant information to aid in understanding complex cases.

## Abbreviations

CI =: confidence interval
EZ =: epileptogenic zone
HFA =: high frequency activity
HFO =: high frequency oscillation
PCA =: principle component analysis
pHFA =: pathological high frequency activity
qHFO =: HFO event detected using the quality-HFO detector
RV =: resected volume
SOZ =: seizure onset zone
